# Calpain Inhibition Promotes the Rescue of F^508^del-CFTR in PBMC from Cystic Fibrosis Patients

**DOI:** 10.1371/journal.pone.0066089

**Published:** 2013-06-13

**Authors:** Monica Averna, Marco Pedrazzi, Laura Minicucci, Roberta De Tullio, Federico Cresta, Franca Salamino, Sandro Pontremoli, Edon Melloni

**Affiliations:** 1 Department of Experimental Medicine (DIMES) - Biochemistry Section, and Center of Excellence for Biomedical Research (CEBR), University of Genoa, Genoa, Italy; 2 Cystic Fibrosis Pediatric Center, G. Gaslini Hospital, Department of Neuroscience, Rehabilitation, Ophthalmology, Genetics and Science Mother and Child, University of Genoa, Genoa, Italy; University of Texas Health Science Center, United States of America

## Abstract

A basal calpain activity promotes the limited proteolysis of wild type (WT) cystic fibrosis conductance regulator (CFTR), inducing the internalization of the split channel. This process contributes to the regulation in the level of the active CFTR at the plasma membranes. In peripheral blood mononuclear cells (PBMC) from 16 healthy donors, the inhibition of calpain activity induces a 3-fold increase in the amount of active WT CFTR at the plasma membranes. Instead, in PBMC from cystic fibrosis (CF) patients, calpain activity is expressed at aberrant levels causing the massive removal of F^508^del-CFTR from the cell surface. In these patients, the inhibition of such abnormal proteolysis rescues physiological amounts of active mutated CFTR in 90% of the patients (25 over 28). The recovery of functional F^508^del-CFTR at the physiological location, in cells treated with a synthetic calpain inhibitor, indicates that F^508^del-CFTR folding, maturation, and trafficking operate in CF-PBMC at significant rate. Thus, an increase in the basal calpain activity seems primarily involved in the CFTR defect observed in various CF cells. Furthermore, in CF-PBMC the recovery of the scaffolding protein Na^+^/H^+^ exchanger regulatory factor 1 (NHERF-1), occurring following inhibition of the aberrant calpain activity, can contribute to rescue CFTR-functional clusters.

## Introduction

The level of mature and functionally active cystic fibrosis conductance regulator (CFTR) at the plasma membranes is under the control of multiple proteolytic systems [1], [2]. The first proteolytic control occurs in endoplasmic reticulum (ER) and is operated by the ATP-ubiquitin-proteasome system, as a part of the general quality control mechanisms involved in the removal of misfolded proteins [3], [4]. In fact, it is reported that approximately only one over three CFTR molecules can reach the mature form, thus escaping this proteolysis [5]. The fraction of F^508^del-CFTR undergoing degradation in ER seems even higher than that of wild type CFTR, explaining the very low level of the channel detectable at the plasma membranes of CF cells [5]. Then, lysosomal proteases are finally involved in the degradation of the chloride channel internalized following the endocytic recycling [6]–[8].

Our previous observations indicate that a third proteolytic system, represented by the Ca^2+^-dependent calpain/calpastatin system, operating at the inner surface of the plasma membranes, seems involved in CFTR turnover [9]. At difference from the other two proteolytic systems, calpain, the Ca^2+^-dependent protease, cleaves the mature and correctly localized CFTR in two discrete fragments of 100 kD and 70 kD. These two fragments remain associated each other on the membranes, both having a transmembrane domain. The split CFTR is then immediately internalized in endocytic vesicles and slowly digested by lysosomes [9]. As a result, significant amounts of the split CFTR are detectable in non-stimulated human leukaemic T cells (JA3), as well as in human peripheral blood mononuclear cells (PBMC), indicating that a basal calpain activity is involved in this process probably required to regulate the levels of mature CFTR at the plasma membranes [9], [10].

Recently, it has been reported that the elastase released from activated neutrophils induces a limited proteolysis of CFTR in airway epithelial cells through activation of intracellular calpains [11]. Such limited conservative proteolysis catalyzed by calpain could be visualized as a selective regulatory process that controls the number of active CFTR molecules at the plasma membranes. We have also observed that F^508^del-CFTR is degraded by calpain, producing the same fragments obtained by the degradation of the wild type channel, and that the deletion of Phe^508^does not modify the sensitivity of the channel to the protease [10], [12]. In PBMC from cystic fibrosis (CF) patients, the mature F^508^del-CFTR is almost undetectable and levels of the split channel form are higher than those detected in control cells [10], [12]. These observations indicate that in cells of CF patients the CFTR defect at the plasma membranes could be attributed, in addition to an incorrect folding of the protein [13], also to an increased calpain activity at the plasma membranes [9], [10] promoted by an impaired cellular calcium homeostasis, detected in cells from CF patients [14]–[17]. Moreover, degradation of F^508^del-CFTR could be further increased because activity of calpain is also sustained by a decreased level of its natural inhibitor calpastatin. The susceptibility of wild type CFTR to calpain digestion seems modulated by the association of the channel to its partner proteins in the CFTR-generated functional complexes. In this respect, of particular importance is the effect exerted by the chaperone HSP90 which protects almost completely membrane inserted mature CFTR from calpain digestion; this process is much less effective for F^508^del-CFTR [10], due to a lower affinity of the mutated channel for the chaperone. Thus, the extent of CFTR degradation in cells appears directly dependent on the organization of these functional complexes.

In our studies, PBMC have been selected as cell model due to the observations indicating that a number of lymphocyte functional properties are altered in CF patients [18]–[20]. Indeed, it has been reported that the lack of mature CFTR in CD3^+^ lymphocytes impairs cytokine secretion and hyperinflammatory adaptive immune responses [18] and a defective level of CFTR seems correlated to impairment of lymphocyte bacterial killing capacity [19]. Moreover, accumulation of lymphocytes in the bronchial mucosa of CF patients has also been described [20]. Although CFTR protein was first thought to be mainly expressed in epithelial tissues, all these findings suggest an important role of CFTR in lymphocytes physiology [21].

In the present study, we are reporting that intracellular calpain inhibition promotes a large increase of CFTR associated to the plasma membranes in PBMC from healthy subjects. Rescue of the channel protein was obtained also in PBMC from CF patients following intracellular calpain inhibition. The recovered F^508^del-CFTR resulted functionally active at an extent comparable to that of the wild type channel. Our observations indicate that, in condition of calpain inhibition, F^508^del-CFTR could acquire the mature active form reaching its correct localization at the plasma membranes, although in an amount significantly lower as compared to the wild type channel form.

The present report not only provides new understanding of the mechanism involved in CFTR trafficking, but also suggests new approaches in the therapeutic strategies for cystic fibrosis.

## Materials and Methods

### Reagents and Antibodies

Leupeptin, CI-2 (calpain inhibitor 2), N^6^,O^2’^-dibutyryl adenosine 3′:5′-cyclic monophpshoric acid (dibutyryl-cAMP) and genistein were purchased from Sigma-Aldrich. 4-(2-aminoethyl) benzenesulfonylfluoride (AEBSF) was obtained from Calbiochem. ECL ADVANCE® Detection System was obtained from GE Healthcare. t-BOC (t-butoxycarbonyl)-Leu-Met-CMAC (7-amino-4-chlorometylcoumarin), a fluorogenic calpain substrate was purchased fromMolecular Probes (Invitrogen). Ficoll-Pacque Plus was obtained from GE Healthcare. Anti -CFTR monoclonal antibody (clone M3A7) was purchased from Millipore. Anti-CFTR polyclonal antibody was purchased from Cell Signaling. Anti-NHERF-1 (H-100) polyclonal antibody was purchased from Santa Cruz biotechnology, Inc. CFTR inhibitor CFTR(inh)-172 [22] was kindly provided by Dr. C. Sorio, Department of Pathology and Diagnosis, University of Verona, Verona, Italy.

### Ethics Statement

All participants and the authorized parents gave written informed consent prior to inclusion in the study, including permission to store the samples and to use them for research exclusively. The study protocol conforms to the provisions of the Declaration of Helsinki and of G. Gaslini Children Hospital, Genoa, Italy. The approval from the Ethics Committees is not required since our analysis were carried out on blood samples obtained from all participants during their routine clinical examinations at the hospital. The acquisition and analysis of the data were anonymous.

### Donor Subjects and Sample Collection

28 CF patients homozygous for F^508^del-CFTR mutation and 16 healthy donors were enrolled in the study. CF patients (15 males; mean age: 32, range: 11÷56, mean FEV1% predicted value for height, sex and age 61%, range: 30÷92; 20/22 with pancreatic insufficiency) were regularly followed at the Cystic Fibrosis Center, G. Gaslini Hospital, Genoa, Italy and blood samples were collected under the supervision of Dr. L. Minicucci. For every patient and healthy donor, a sample of 6 ml of blood was collected in two 3 ml vacuette® PREMIUM tubes containing 5 mM EDTA. Samples were rapidly chilled in an ice bath and transferred to the DIMES, Section of Biochemistry, for the experimentation.

### Incubation of PBMC with CI-2 and Isolation of the Total Membrane Fraction

PBMC, isolated as previously described [23], were washed twice with RPMI1640 growth medium, containing 10% FBS, 10 U/mL penicillin, 100 µg/mL streptomycin, 4 mM L-glutamine (culture medium), plated at 5×10^6^ cells/flask and incubated for 24 h at 37°C in culture medium supplemented with the vehicle (DMSO, 0.1 µL/mL) or the amounts of CI-2 indicated elsewhere in a humidified atmosphere containing 5% CO_2_. Viability as assessed by 0.2% trypan blue exclusion was higher than 80–90%. After incubation, cells were collected by centrifugation at 800×g for 10 min. PBMC were washed three times with ice-cold PBS solution and suspended in 50 mM sodium borate, pH 7.5 (500 µL), containing 1 mM EDTA, 2.5 µg leupeptin and 5 µg AEBSF. Cells were lysed by three cycles of freezing and thawing followed by sonication. The total particulate fraction was collected by centrifugation at 100,000 g for 10 min at 4°C. The pellet (total membrane fraction) was washed three times in 50 mM sodium borate, 0.1 mM EDTA, pH 7.5 and finally suspended in 0.1 mL of the washing buffer. The protein concentration was determined following the method of Lowry.

### Immunoblotting

Total membrane fraction (10 µg), prepared from PBMC as described above, was incubated at 37°C for 30 min in Laemmli loading buffer and submitted to 6% SDS-PAGE [24]. Proteins were then transferred to nitrocellulose membranes (Bio-Rad) by electroblotting [25] and probed with the specific antibodies, followed by a peroxidase-conjugated secondary antibody as described in [26]. The immunoreactive material was developed with ECL ADVANCE® detection system, detected with a Bio-Rad Chemi Doc XRS apparatus, and quantified using the Quantity One 4.6.1 Software (Bio-Rad Laboratories). The values obtained were always normalized using beta-actin as loading control.

### Isolation of the Plasma and Intracellular Membranes

Total membrane fraction (100 µg in 0.5 ml) prepared from untreated and CI-2 treated PBMC was fractionated on a discontinuous sucrose density gradient [10, 20, 30 and 50% (w/v)]. After centrifugation at 38000 rpm in a Beckman Coulter TLA-100.4 rotor for 30 min, membranes were recovered into two fractions layered at 10% and 30% sucrose interface. They were separately collected, suspended in 0.2 ml of sodium borate 50 mM, pH 7.5 containing 1 mM EDTA and 5′-nucleotidase (5′-NT) activity was assayed [27], as plasma membranes marker. In the same samples protein concentration following Lowry method was also determined.

### Confocal Microscopy Imaging

PBMC (1×10^7^) isolated from control and CF patients were fixed and permeabilized by the Triton/paraformaldehyde method, as described in [28]. Cells were treated with 10 µg/mL CFTR antibody (M3A7) diluted in PBS solution, containing 5% (v/v) FBS. After incubation for 3 h at 25°C, cells were washed three times with PBS solution and treated with 4 µg/mL chicken anti-(mouse IgG) Alexa fluor® 488-conjugate secondary antibody (Molecular Probes) for 1 h. Chromatin was stained by exposing fixed and permeabilized cells to 2 µg/mL propidium iodide for 5 min [29]. Images were collected using a Bio-Rad MRC1024 confocal microscopy, with a 60× Plan Apo objective with numerical aperture 1.4. The excitation/emission wavelengths for propidium iodide-stained chromatin were 568/605 nm. Sequential acquisitions were performed to avoid cross-talk between colour channels.

### Assay of Intracellular Calpain Activity

Calpain activity was detected in PBMC from healthy donors or CF patients following the procedure described in [30]. Briefly, PBMC (1×10^6^ cells/mL) were collected, washed three times in PBS solution, and incubated for 20 min at 37°C in buffer A (10 mM Hepes, pH 7.4, 0.14 M NaCl, 5 mM KCl, and 5 mM glucose) containing 50 µM t-Boc-Leu-Met-CMAC, the fluorogenic calpain substrate. Cells were washed with buffer A to remove substrate excess. The pellets were suspended to give 10^6^ cells/mL in buffer A containing 0.1 mM CaCl_2_ and different concentrations (from 0 to 2 µM) of CI-2. Aliquots (2×10^5^ cells) were transferred to 96-well plates and the fluorescence emission was continuously monitored at 37°C for 2 hours with a Mithras LB940 plate reader (Berthold Technologies). The excitation/emission wavelengths were 355/485 nm respectively.

### Isolation of the Halide-sensitive Yellow Fluorescent Protein (HS-YFP)

Fischer Rat Thyroid (FRT) cells, stably transfected with F^508^del-CFTR and halide-sensitive yellow fluorescent protein YFP-H148Q/I152L (HS-YFP) [31], were provided by Dr L. J. Galietta, Molecular Genetics Laboratory, G. Gaslini Hospital, Genoa, Italy. HS-YFP was purified following the procedure previously described in [32]. HS-YFP fluorescence was 50% reduced by addition of 1.78±0.08 mM NaI or 86±1.12 mM NaCl.

### Assay of CFTR Activity

Measurements of CFTR activity were carried out on PBMC isolated from healthy donors (controls) and CF patients incubated for 24 hours in the absence or presence of 2 µM CI-2. Cells (1×10^6^), washed twice with 0.25 M sucrose, containing 20 mM sodium borate, pH 7.5, and 0.2 mM Ca(NO_3_)_2_ (buffer CFTR), were suspended in 100 µL of buffer CFTR and exposed for 30 min at 37°C to vehicle or 100 µM dibutyryl-cAMP and 50 µM of the CFTR potentiator genistein [33]. Afterwards, 5 mM NaI was added to the cell suspensions and maintained for 20 seconds. Following that, cells were discarded by centrifugation at 13,000×g for 20 seconds and the clear supernatants were collected. Aliquots of the supernatants (90 µL) were placed in 96-well plates and 1 µg of purified HS-YFP was added. Fluorescence was measured with a Mithras LB940 plate reader (Berthold Technologies); the excitation/emission wavelengths were 485±15/535±10 nm, respectively. CFTR activity was assayed based on the decrease in extracellular I^-^ concentration measured by means of the increase in HS-YFP fluorescence, with respect to the fluorescence evaluated in the presence of 5 mM NaI.

## Results

### Calpain Inhibition Promotes Accumulation of Mature CFTR in Control-PBMC

**Figure 1:**
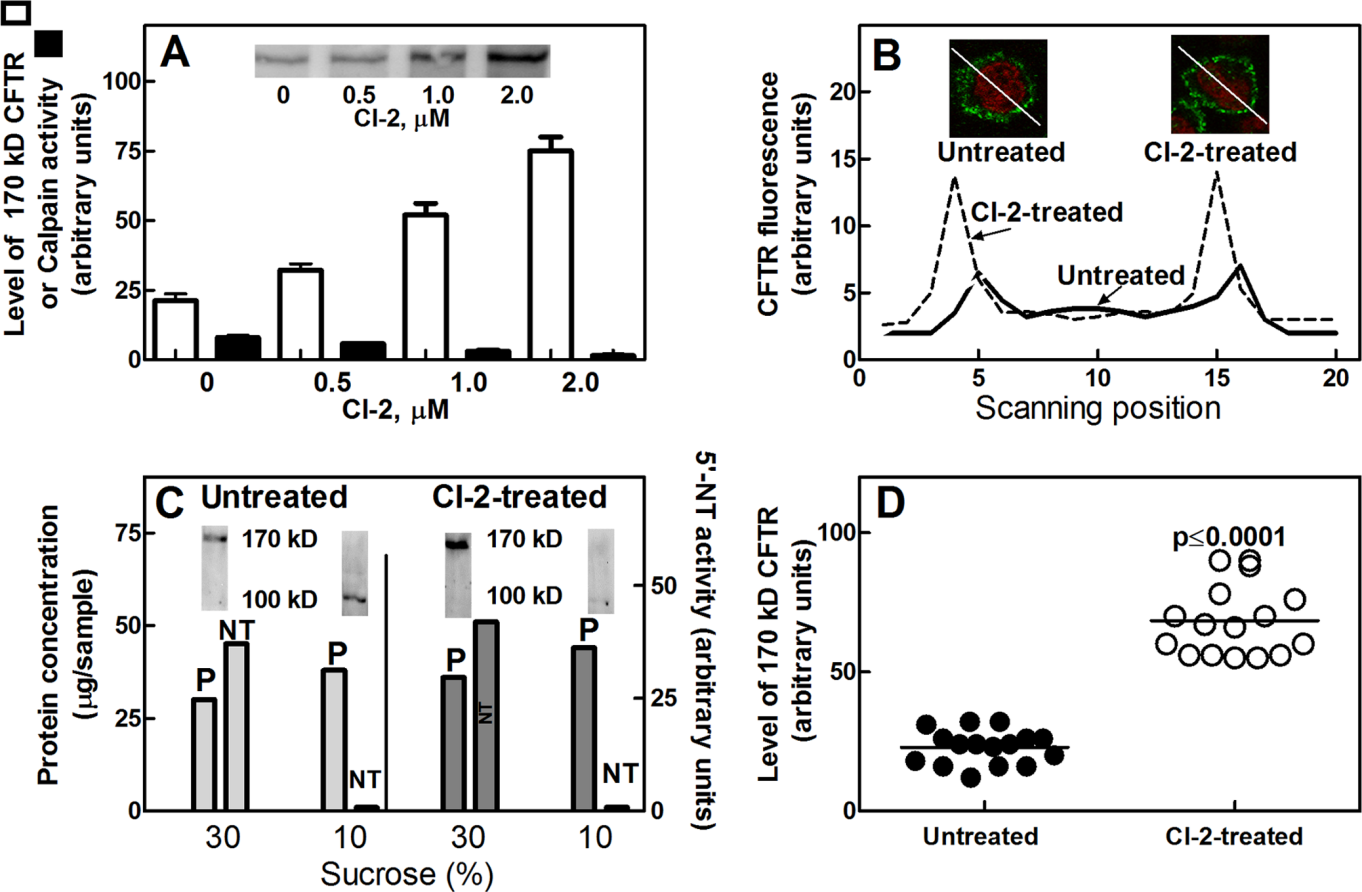
Levels of 170 kD CFTR in control-PBMC treated with CI-2. (**A**) Control-PBMC (1×10^6^) were incubated in the absence or presence of the indicated concentrations of CI-2 for 24 hours. Total membrane fraction (10 µg of protein) was submitted to 6% SDS-PAGE followed by immunoblotting. Unfilled bars represent the quantification of mature 170 kD CFTR immunoreactive signals; a representative blot is shown in the inset. Filled bars represent intracellular calpain activity. The values reported are the arithmetic mean ± SD of three different quantifications performed on cells from three healthy donors. (**B**) Cells (1×10^7^) untreated or treated with 2 µM CI-2 for 24 hours were fixed and CFTR localization was determined by confocal microscopy. Nuclei were stained with 2 µg/ml propidium iodide for 5 min [29]. CFTR signal (green fluorescence) was continuously monitored during cell scanning (from left to right following the white line) by using Laser Pix software. Each scanning trail is representative of 20 cells analyzed. (**C**) Control-PBMC (1×10^7^) were incubated in the absence or presence of 2 µM CI-2 for 24 hours. Cell membranes recovered from 10% and 30% sucrose interface were used to detect CFTR by immunoblotting, to assay 5′-nucleotidase activity (NT), and to measure protein content (P). (**D**) Quantification and statistical analysis of 170 kD CFTR levels were carried out in PBMC from 16 healthy donors. PBMC were untreated or treated with 2 µM CI-2 for 24 hours. Statistical analysis with ANOVA test for a single factor revealed that the 95% confidence intervals of the different analysis did not superimpose showing a p-value≤0.0001.

We have recently reported that degradation of CFTR in condition of basal calpain activity is detectable in growing cells as well as in PBMC. When calpain activity is evoked by a rise in [Ca^2+^]_i_, CFTR cleavage goes to completion resulting in the removal of the channel from the plasma membranes [10]. On the basis of all these information, we have now explored if, following reduction of intracellular calpain activity, CFTR undergoes accumulation in a mature and active form. Thus, PBMC isolated from healthy donors were incubated for 24 hours at 37°C (5% CO_2_) in the presence of increasing concentrations (from 0 to 2 µM) of CI-2, a synthetic calpain inhibitor interacting with the essential Cys residue at the protease active site [34]. As shown in Fig. 1A, although in untreated PBMC calpain activity is very low, it is further reduced in the presence of increasing CI-2 concentrations. In this condition, the amount of mature 170 kD CFTR resulted 3-fold higher at 2 µM CI-2. These findings indicate that a basal limited calpain activity is involved in the physiological control of mature CFTR level. Confocal microscope inspection (Fig. 1B) revealed that, in cells exposed to CI-2, the accumulation of CFTR occurred at the cell surface and, as observed by scanning of the images, CFTR fluorescence at the cell periphery was approximately 3-fold higher than in untreated cells. These findings suggest that the accumulated CFTR molecules have reached their functional localization at the plasma membranes. To further verify this hypothesis, both the plasma- and internal-membrane fractions from CI-2-treated cells were separated by a sucrose density gradient. Following centrifugation, two membrane fractions were collected: the first one layered at 10% sucrose interface and the second one, that layered at the 30% sucrose, contained 5′-nucleotidase activity, thus corresponding to the plasma membranes. As shown in Fig. 1C, mature 170 kD CFTR was exclusively detected in the plasma membrane fractions. Moreover, the calpain digested 100 kD CFTR form, well represented in untreated cells, becomes poorly detectable in cells treated with CI-2, indicating that internalized split CFTR is further degraded by other proteolytic systems [35]. It is important to note that the accumulation of mature CFTR, observed in PBMC exposed to CI-2, was detected in all the 16 healthy donors tested (Fig. 1D) with a mean increase at the plasma membranes of approximately 3 times. Since all samples were responsive to calpain inhibition, it can be concluded that basal calpain activity is required to regulate the level of mature CFTR at the cell surface.

### Calpain Inhibition Promotes Accumulation of Mature F^508^del-CFTR in PBMC from CF Patients at the Plasma Membranes

**Figure 2:**
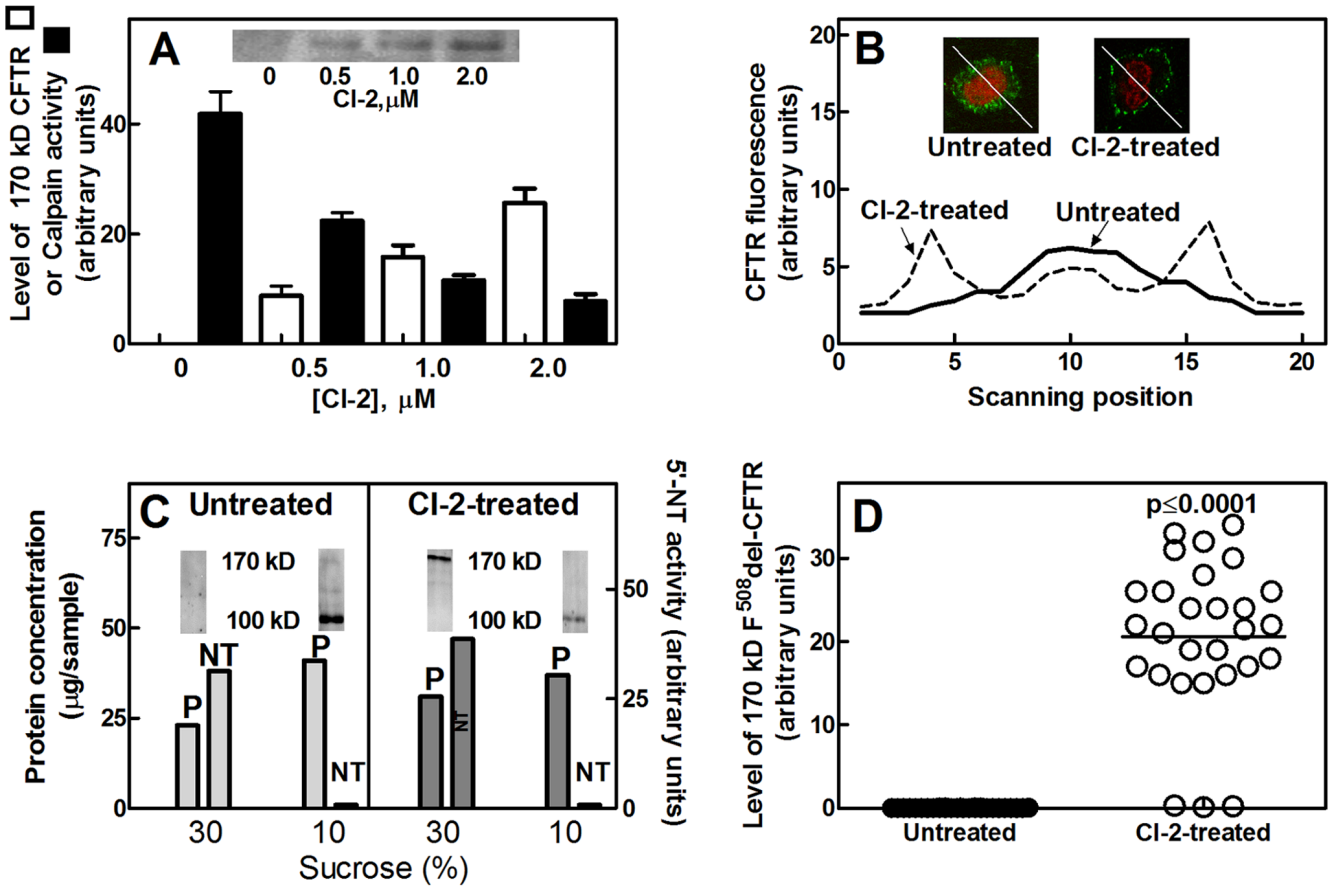
Levels of 170 kD F^508^del-CFTR in CF-PBMC treated with CI-2. (**A**) CF-PBMC (1×10^6^) were incubated in the absence or presence of the indicated concentration of CI-2 for 24 hours. Total membrane fraction (10 µg of protein) was submitted to 6% SDS-PAGE followed by immunoblotting. Unfilled bars represent the quantification of mature 170 kD CFTR immunoreactive signals; a representative blot is shown in the inset. Filled bars represent intracellular calpain activity. The values reported are the arithmetic mean ± SD of three different quantifications performed on cells of three patients. (**B**) Cells (1×10^7^) untreated or treated with 2 µM CI-2 for 24 hours were fixed and CFTR localization was determined by confocal microscopy. Nuclei were stained with 2 µg/ml propidium iodide for 5 min [29]. CFTR signal (green fluorescence) was continuously monitored during cell scanning (from left to right following the white line) by using Laser Pix software. Each scanning trail is representative of 20 cells analyzed. (**C**) CF-PBMC (1×10^7^) were incubated in the absence or presence of 2 µM CI-2 for 24 hours. Cell membranes recovered from 10% and 30% sucrose interface were used to detect CFTR by immunoblotting, to assay 5′-nucleotidase activity (NT), and to measure protein content (P). (**D**) Quantification and statistical analysis of 170 kD CFTR levels were carried out in PBMC from 28 patients. PBMC were untreated or treated with 2 µM CI-2 for 24 hours. Statistical analysis with ANOVA test for a single factor revealed that the 95% confidence intervals of the different analysis did not superimpose showing a p-value≤0.0001.

We have recently reported that mature F^508^del-CFTR is nearly absent in PBMC from CF patients, suggesting an increased ER degradation of the misfolded channel by the proteasome machinery [10], [12]. However, on the basis of the previous and present observations it can be assumed that an increased calpain activity is involved in the massive removal of F^508^del-CFTR from the plasma membranes. Thus, PBMC from CF patients were exposed for 24 hours to increasing concentrations of CI-2 and the localization as well as the amount of mature F^508^del-CFTR were evaluated. As shown in Fig. 2A, while in untreated CF-PBMC 170 kD F^508^del-CFTR was not detected, calpain activity was 5-fold higher than that observed in cells from healthy donors (see Fig. 1A). When PBMC from CF patients were exposed to increasing amounts of CI-2, we observed that the 170 kD F^508^del-CFTR was progressively accumulated reaching, in the presence of 2 µM CI-2, a protein amount similar to that found in untreated control cells. These data indicate that an aberrant calpain activity is responsible for the channel defect at the plasma membranes. Confocal microscope images (Fig. 2B) revealed that, following CI-2 treatment, also in CF-PBMC the CFTR fluorescence was detected as a ring around the cells, suggesting the accumulation of mature 170 kD channel form at the plasma membranes. Notably, we also confirmed that, following membrane separation on a sucrose density gradient (Fig. 2C), rescued 170 kD F^508^del-CFTR was exclusively associated to the plasma membrane fraction. Concomitantly, the amount of calpain-cleaved 100 kD F^508^del-CFTR, the only channel form detectable in untreated CF-PBMC (Fig. 2C), was largely reduced due to a decreased rate of formation and to a further digestion by lysosomes [36], [37]. These data confirm our initial hypothesis that in CF-PBMC calpain over-activation promotes the removal of the mutated channel from the plasma membranes. As shown in Fig. 2D, the inhibition of calpain activity leads to the accumulation of 170 kD F^508^del-CFTR in PBMC from 25 of the 28 CF patients analyzed. Thus, 90% of the patients are responsive to CI-2 treatment and the mean value of the rescued F^508^del-CFTR (22 units) is similar to that observed in untreated PBMC from healthy subjects (24 units), confirming the results shown in Fig. 2A.

### Calpain Inhibition Promotes Rescue of Active CFTR in Control- and CF-PBMC

**Figure 3:**
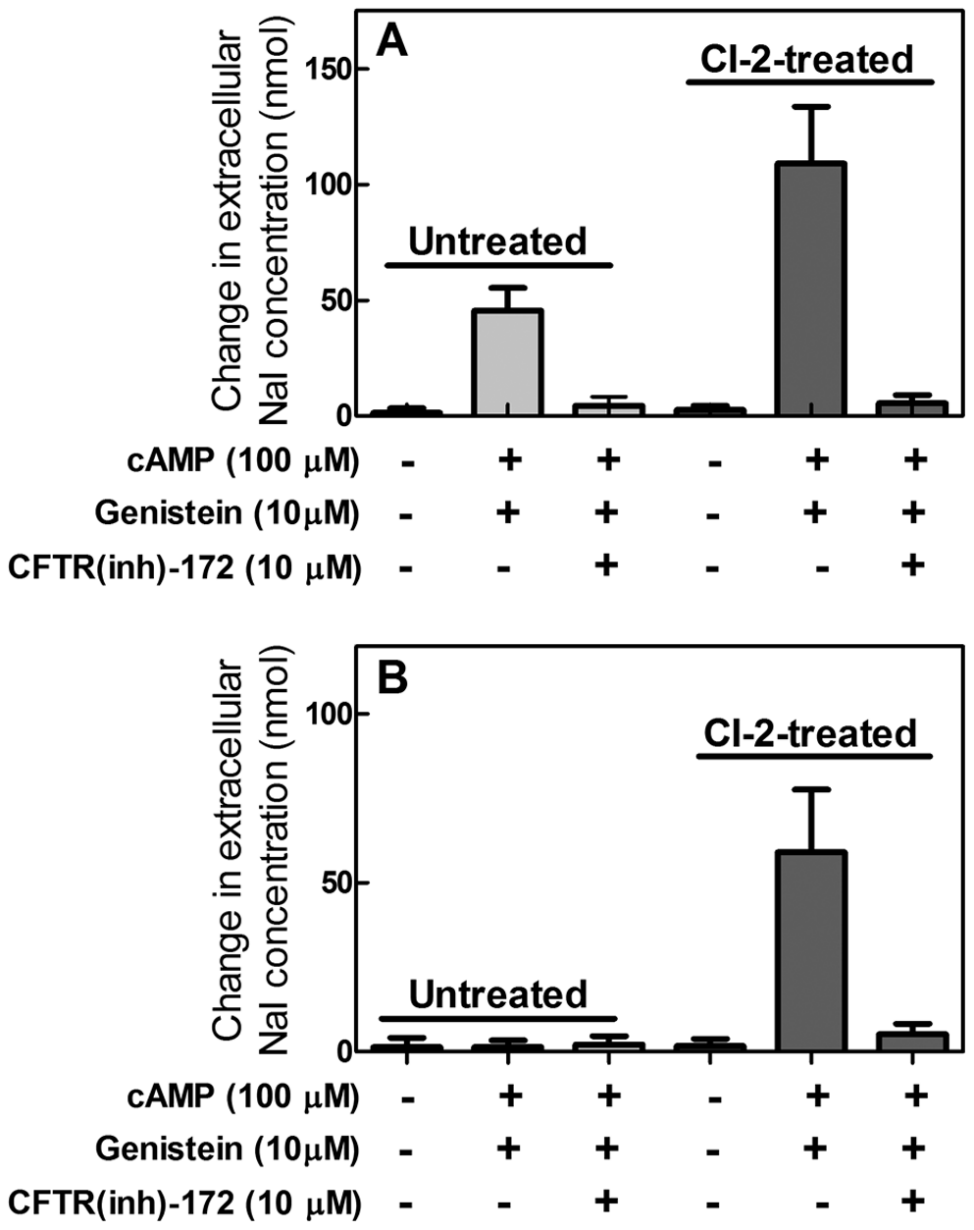
Levels of CFTR activity in PBMC from controls and CF patients treated with CI-2. (**A**) Control-PBMC and (**B**) CF-PBMC were treated in the absence or presence of 2 µM CI-2 for 24 hours. To detect specific CFTR activity cells were exposed to the indicated stimuli. The values are the arithmetic mean ± SD of three different evaluations performed on cells of five patients and correspond to the difference between the initial minus the final extracellular NaI nmoles measured in the conditions reported.

Even though our observations clearly indicate that calpain inhibition promotes the accumulation of CFTR at its functional localization, the assay of CFTR activity is of great relevance, since it represents a direct indication that the recovered channel is present in its functional conformational state. To this purpose, the CFTR-mediated iodide flux across the plasma membranes was evaluated in untreated and in CI-2-treated PBMC from both healthy donors and CF patients. Our assay method is based on the activation of CFTR by cAMP-dependent phosphorylation and the measurement of iodide influx into the cells. The iodide concentration in the extracellular medium was determined by means of the fluorescent HS-YFP [31]. In addition, genistein, a well known CFTR potentiator [33], was added to favour the opening of the channel pore in order to obtain fluxes more proportional to CFTR amounts. As shown in Fig. 3A, in untreated control-PBMC exposed to cAMP and genistein, CFTR activity was 2.5-2.8-fold increased in cells previously incubated with 2 µM CI-2. The iodide influx was blocked in both untreated and treated cells by the addition of the CFTR (inh)-172, indicating that the decrease in extracellular iodide concentration is specifically due to CFTR activity. It is important to note that the rise in the CFTR activity induced by CI-2 treatment is consistent with the accumulation of the mature protein channel at the plasma membranes, occurring in the same conditions (see Fig. 1A).

To establish if also the F^508^del-CFTR rescued in CI-2 treated CF-PBMC is active, the activity of the mutated channel was measured in the same conditions used in control-PBMC isolated from healthy donors. As shown in Fig. 3B, CFTR activity, undetectable in untreated PBMC from CF patients, becomes measurable at high levels in CI-2 treated cells. The iodide influx was prevented by CFTR (inh)-172, confirming that the anion entry is specifically mediated by the mutated F^508^del-CFTR present in these cells. These data are in agreement with the report indicating that in the presence of cAMP and genistein the open probabilities of WT and F^508^del-CFTR are identical [38]. The recovery of the channel activity also indicates that F^508^del-CFTR is able to reach the plasma membranes in a mature and active form.

**Figure 4:**
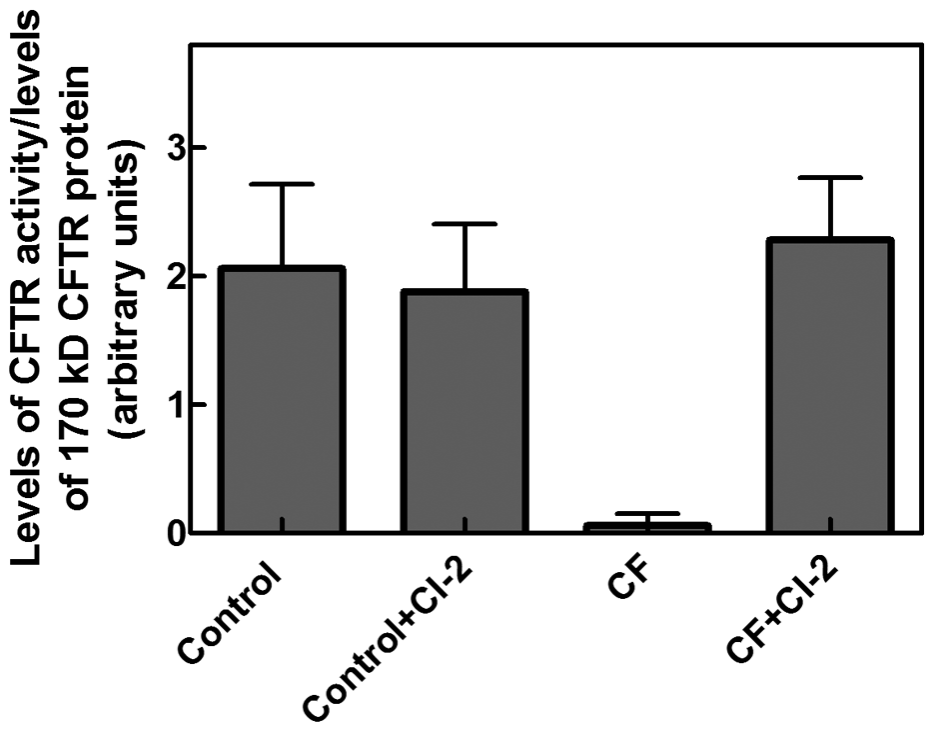
Ratio between the level of CFTR activity and the intensity of the 170 kD protein band in PBMC from controls and CF patients treated with 2 µM CI-2. CFTR activity was determined as described in Material and Methods. The levels of the mature 170 kD CFTR protein in control and CF-PBMC were obtained by scanning blots carried out as those shown in Figure 1 and 2. The values are the arithmetic mean ± SD of three different analyses.

Moreover, calculation of the ratio between the level of channel activity and the intensity of the 170 kD protein band revealed that the specific activity of WT CFTR is similar to that of the F^508^del-CFTR rescued in CF-PBMC exposed to CI-2 (Fig. 4). It is interesting to note that the amount of CFTR rescued in condition of calpain inhibition seems higher than that previously obtained with compounds affecting the rate of channel folding and maturation [39], [40]. This observation points out that the aberrant calpain activity detected in CF-PBMC is involved in the defect of CFTR at the plasma membranes. This conclusion is further supported by the fact that approximately only one third of the potential newly synthetized F^508^del-CFTR molecules can reach maturation and the functional localization [41].

### Effect of Calpain Inhibition on CFTR Partner Proteins

**Figure 5:**
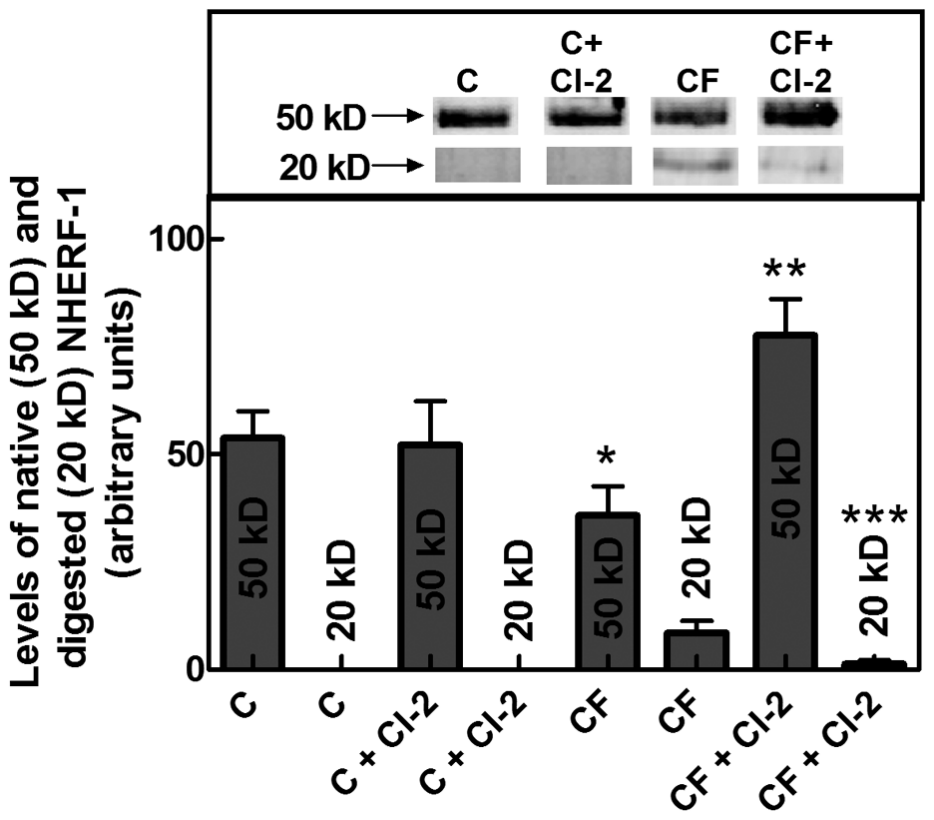
Levels of NHERF-1 in PBMC from controls and CF patients treated with CI-2. PBMC (2.5×10^4^), untreated or treated with 2 µM of CI-2 (CI-2) for 24 hours, obtained from 7 healthy donors (C) and 10 CF patients (CF), were submitted to 12% SDS-PAGE followed by immunoblotting. Native (50 kD) and digested (20 kD) NHERF-1 immunoreactive signals were quantified, and statistical analysis by ANOVA, followed by post hoc Tukey’s test, was carried out. Representative blot images for the two NHERF-1 forms are shown in the upper panel. *Significantly different from C (50 kD) p<0,05; **significantly different from CF (50 kD) p<0,001; ***significantly different from CF (20 kD) p<0,001.

To establish if, in addition to CFTR, the CFTR partner protein NHERF-1, known to undergo digestion by calpain in CF-PBMC [10], is also recovered in CI-2-treated cells, the levels of the native 50 kD and the calpain-digested 20 kD NHERF-1 forms were comparatively analyzed in control and CF-PBMC. As shown in Fig. 5, in cells from healthy donors the native 50 kD species was the only NHERF-1 form detectable and its level was not significantly modified following CI-2 treatment. In untreated CF-PBMC, we observed that the native NHERF-1 form was 20-30% reduced (p<0,05) as compared to control, whereas the calpain-digested 20 kD NHERF-1 form was 10-15% increased. Following exposure to CI-2, this degraded form was 80% reduced (p<0,001) while native NHERF-1 was approximately 2-fold raised (p<0,001). The increased levels of native NHERF-1 together with the disappearance of NHERF-1 digested form, observed in these experimental conditions, are in accordance with the extent of calpain inhibition exerted by CI-2 (see Fig. 2A). Since NHERF-1 favours the recovery of CFTR at the plasma membranes [42], the increase in native NHERF-1 levels, observed in CF-PBMC following intracellular calpain inhibition, could further enhance the rate of the channel accumulation at the plasma membranes.

## Discussion

We are herewith reporting that WT CFTR is 3-fold accumulated at the plasma membranes in an active form following intracellular inhibition of calpain activity. This CFTR rescue has been observed in PBMC from all the subjects analyzed, indicating that this process is of general significance. In fact, our findings indicate that a basal calpain activity, constitutively operating in PBMC, promotes the cleavage of WT CFTR and its removal from the plasma membranes. This process represents a physiological calpain function that is required to regulate the level of the active channel species, since calpain-mediated digestion of CFTR occurs only at the plasma membranes. In PBMC from CF patients, that are characterized by an aberrant calpain activity, F^508^del-CFTR is absent from the plasma membranes being the split form largely accumulated inside the cells. Instead, when calpain activity was inhibited by CI-2, we observed the recovery of F^508^del-CFTR at the plasma membranes up to physiological amounts. In fact, following calpain inhibition, the level of the rescued mutated channel becomes similar to that of the WT CFTR expressed in PBMC from healthy donors. The effectiveness of such treatment indicates that calpain-mediated proteolysis of CFTR plays a crucial role in promoting the channel defect observed in CF cells. It is interesting to note that treatment with various effectors, operating in CFTR maturation steps [39], [40], seems unable to induce the accumulation of comparable amounts of the channel at the plasma membranes. Notably, we also demonstrate that once F^508^del-CFTR has reached its appropriate localization it is biologically active. This fact indicates that the specific steps involved in processing, maturation, and trafficking of CFTR occur also in CF cells, although at lower rate. Thus, the limited efficiency of correctors could be due to their inability to reduce the aberrant calpain activity operating at the plasma membranes. In this respect, a recent paper [11] reports that in CF-airway cells the elastase released by activated neutrophils is involved in the abnormal activation of calpain. Although these data suggest a mechanism leading to calpain activation, such process can operate only in response to bacterial infections. It is very important to note that PBMC from 90% of the analyzed CF patients resulted responsive to CI-2 treatment and only in three patients, F^508^del-CFTR was not recovered at significant levels. At present it is difficult to explain these negative results; it is possible that they may be due to the multiple therapeutic treatments at which these patients are exposed. Moreover, the level of NHERF-1, a CFTR partner protein known to be a calpain substrate, is increased in CF-PBMC following calpain inhibition. It has been reported [42] that overexpression of this scaffolding protein favours the rescue of F^508^del-CFTR at the plasma membranes. This observation is in agreement with our present results showing that the recovery of NHERF-1 can enhance the organization of CFTR functional clusters at the cell surface.

These observations indicate that the calpain proteolytic system could be a promising target for CF therapy. However, since appropriate compounds are not available at present, experiments are in progress in our laboratory to obtain suitable inhibitors based on calpastatin structure.
